# Influence of Particle Size and Mineralogical Composition on the Mechanical and Tribological Properties of Resin-Regolith-Composites for Non-Structural Applications

**DOI:** 10.3390/ma19102066

**Published:** 2026-05-15

**Authors:** Nicola Calisi, Stefano Caporali, Rosa Taurino

**Affiliations:** 1 Department of Industrial Engineering (DIEF), University of Florence (UNIFI), Via di Santa Marta n. 3, 50139 Firenze, Italy; rosa.taurino@unifi.it; 2 National Interuniversity Consortium of Materials Science and Technology (INSTM), Via Giuseppe Giusti n. 9, 50121 Firenze, Italy

**Keywords:** composite, epoxy resin, lunar regolith simulant, particle size, mechanical properties

## Abstract

The development of resin-regolith composites represents a promising In Situ Resource Utilization (ISRU) strategy for future lunar missions. While unsuitable for primary habitat construction due to the payload cost of transporting polymers from Earth, these composites offer a highly efficient solution for manufacturing non-structural, everyday items (e.g., containers, tools, and plant cultivation pots) directly on the Moon via mold–casting. This approach significantly reduces the volume and mass of pre-formed plastic payloads. In this work, the influence of the particle size distribution of a lunar highland simulant (LHS-1E) on the mechanical properties of epoxy-based composites was systematically investigated for such applications. First, the regolith-to-resin ratio was optimized for castability, establishing a maximum regolith content of 60 wt.%. Then, four different size fractions of the simulant were prepared by sieving (>200 µm, 200–100 µm, 100–50 µm, and <50 µm), and composite samples were cast maintaining this optimal ratio. X-ray microtomography revealed that using larger particles (>200 µm) increased composite porosity, whereas smaller fractions promoted more compact structures. Three-point bending tests showed that intermediate particle sizes (200–100 µm and 100–50 µm) led to enhanced flexural strength, while the smallest particles (<50 µm) decreased mechanical performance, likely due to a lower basalt content in this finer fraction. Finally, ball-on-disk tribological analyses highlighted that composites made with larger particles (>200 µm) exhibited superior wear resistance, whereas particle size had negligible effects on the coefficient of friction. Overall, the results demonstrate that both particle size and mineralogical composition significantly influence the performance of regolith–epoxy composites, providing essential guidelines for the in situ manufacturing of functional, non-structural objects for lunar outposts.

## 1. Introduction

The vision of deep space exploration and the establishment of permanent lunar settlements has made the development of technologies based on In Situ Resource Utilization (ISRU) and On-Site Manufacturing (OSM) crucial. The primary goal of this shift is to minimize costs and reduce dependency on raw materials supplied from Earth, given the immense expense associated with transport [1,2]. Regolith consists of a fragmented layer of unconsolidated material that blankets the surfaces of rocky planetary bodies such as the Moon, Mars, and asteroids. On the Moon’s surface the zone of heavily fractured material affected by intense impact bombardment early in the history of the Solar System extends kilometers deep [3]. Superficial regolith is widely recognized as the most available resource on the Moon and offers crucial advantages in the space environment, including its sheer abundance, resistance to extreme temperature cycles, and intrinsic potential as a radiation-shielding material [4].

The properties of lunar regolith are intrinsically linked to its geological origin and maturity. Mature regolith, which has undergone prolonged exposure to space weathering, typically exhibits a finer grain size and a higher concentration of glassy phases and nanophase iron (nFe^0^) [5]. Mineralogically speaking, regolith is primarily composed of oxides and silicates, comprising approximately 43% oxygen, 21% silicon, and 13% iron, along with various minor elements. Based on its chemical and mineralogical makeup, two main types of regolith are distinguished [6]:

- Mare Regolith: Characterized by the prevalence of basalts and high concentrations of iron (Fe) and titanium (Ti).
- Highland Regolith: Dominated by anorthosite, featuring elevated levels of aluminum (Al) and calcium (Ca).

Regarding morphological properties, lunar regolith particles are characterized by irregular shapes and sharp, angular edges. This unique morphology is a direct result of space weathering processes occurring in the absence of an atmosphere [6]. This heterogeneity is caused by millions of years of meteoritic bombardment and continuous interaction with the solar wind [7]. Specifically, regolith particles exhibit a wide variety of shapes, typically described as angular, vesicular, and often agglutinated [8]. For instance, the simulant EAC-1A is known for its irregular shapes and sharp edges, features highly comparable to those of actual lunar soil [9]. Regolith is typically very poorly sorted, containing particles that span several orders of magnitude in size. It is predominantly fine-grained, with most particles smaller than 1 cm and average grain sizes commonly between 40 and 100 μm, classifying it as a polydisperse powder. Particle size is strongly influenced by the maturity of the material: mature regolith, having experienced prolonged exposure to space weathering, generally consists of finer grains, whereas immature regolith is characterized by coarser particles and larger pore structures [5].

For many applications, it may be beneficial—or even necessary—to separate the material into distinct fractions. In terrestrial mineral processing, sieving methods are the most basic way to sort a mixture of grains. However, gravity-driven sieving could be ineffectual in extraterrestrial low-gravity environments. In such environments it could be possible to achieve size partitions by means of centrifugal sieving [10].

Recently, research has focused on the development of regolith–polymer composites (RPCs), often referred to as “lunar polymer concrete” or “Regolith-Resin Composites” (RRCs). The use of polymers as binding agents offers several key benefits, including ease of fabrication, structural stability, and good thermal resilience. Introducing a polymeric phase is an effective strategy to bond regolith particles and produce solid, lightweight composites, while significantly reducing the energy consumption associated with high-temperature processing methods [5].

Several studies investigated the development of RRCs for large-scale lunar construction applications [4]. Both thermosetting matrices (such as epoxy resins) and thermoplastic matrices have been explored, with the aim of producing strong materials with high mechanical performance and good thermal resistance. For example, Tafsirojjaman et al. [4] developed and characterized an RRC-based on epoxy resin and the lunar highland simulant LHS-1. The material, containing 20% resin, exhibited superior performance compared to terrestrial polymer concrete, achieving a compressive strength of 98 MPa (vs. 80 MPa), flexural strength of 45 MPa (vs. 23 MPa), and tensile strength of 25 MPa (vs. 13 MPa).

Similarly, Bao et al. [2] developed an epoxy–regolith slurry (using the SC80 simulant) suitable for 3D printing via direct ink writing, demonstrating that a mixture of cementite–sand in a ratio of 1:4 provides optimal printability.

In parallel, inorganic composites have also been investigated. This approach exploits the aluminosilicates in regolith as precursors, which are then activated by alkaline solutions. In this case, materials are fabricated through casting or molding [7]. The method involves dry mixing the regolith simulant with a solid alkaline activator, such as sodium silicate, followed by the addition of water. This is advantageous as it eliminates the risks associated with transporting corrosive liquid reagents [11]. The resulting geopolymers have demonstrated suitability for both radiations shielding (due to their high density) and for infrastructure construction [8,12,13].

These studies highlight how the particle size and distribution of regolith represent critical factors in the successful fabrication of polymer- or geopolymer-based composites. The irregular morphology and sharp edges of regolith particles, also linked to grain size, can act as stress concentrators, potentially reducing the tensile properties of polymer composites [14]. Furthermore, these morphological features significantly impact the material’s overall processability.

While literature primarily focuses on bulk infrastructure, transporting polymer matrices from Earth for primary habitat construction is highly inefficient due to severe payload mass and volume limitations. Conversely, utilizing RRCs for the OSM of non-structural, everyday items—such as tools, plant cultivation pots, and small functional containers—represents a highly strategic and viable application of ISRU. By employing local regolith as a filler in mold—casting processes, the dependency on pre-formed plastic items brought from Earth can be drastically reduced, making the transport of the resin matrix logistically advantageous.

For these reasons, the present research investigates the effects of different regolith particle size distributions on the mechanical and physical properties of epoxy resin-based cast composites tailored for the manufacturing of these small-scale, non-structural objects. After determining the maximum regolith loading achievable in an epoxy matrix to optimize castability, the influence of particle size distribution on the resulting material performance was systematically evaluated.

While this study provides foundational data under terrestrial conditions, the transition to lunar manufacturing introduces several environmental variables. It should be noted, however, that the composites developed in this work are primarily intended for pressurized, climate-controlled environments within lunar bases. In such habitats, the absence of vacuums and the presence of stable temperatures significantly mitigate the risks related to the outgassing and thermal fatigue of the epoxy matrix. A critical remaining factor is the lunar gravity (approx. 1.62 m/s^2^). Reduced gravity would substantially alter the rheological behavior during processing; specifically, the sedimentation rate of the heavier basalt particles would be roughly six times slower than on Earth. This could potentially allow for lower resin viscosities or longer curing times without compromising the homogeneity of the filler distribution. Future studies should include experiments in simulated microgravity or incorporate multiphase flow modeling to fully optimize the casting process for on-site lunar manufacturing.

## 2. Materials and Methods

### 2.1. Materials

The lunar regolith simulant used in this study LHS-1E (Space Resource Technologies, Oviedo, FL, USA) is an engineering-grade simulant representative of the lunar highlands, developed using the average composition of these regions as a reference [15,16]. It consists of 75 wt.% anorthosite and 25 wt.% glass-rich basalt. Its uncompressed bulk density is 1.27 g/cm^3^, the median particle size is 80.86 µm, and the particle size distribution ranges from 0.04 µm to 1000 µm [17].

A two-component epoxy resin (Kerakoll EP21, Sassuolo, Italy), typically used for organic screeds, was employed as the binding matrix. The resin-to-hardener weight ratio was 2.5:1.

### 2.2. Composites Manufacturing

To optimize the composite preparation, the study was carried out in two main steps. In the preliminary phase, composites containing different amounts of regolith (Table 1) were prepared to determine the maximum filler content that could be incorporated into the selected epoxy resin.

**Figure 1:**
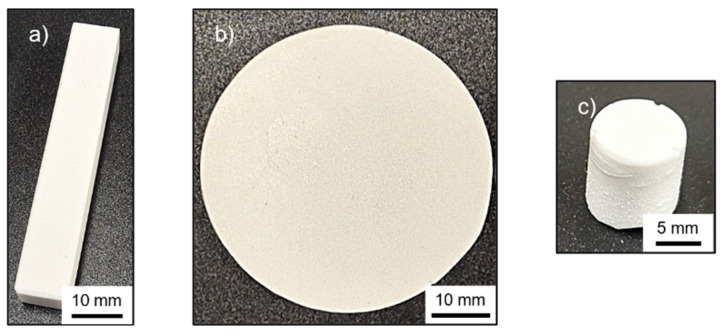
(**a**) Plank-shaped, (**b**) disk-shaped, and (**c**) cylindrical-shaped samples.

The results of this phase were then used to define the optimized regolith concentration and to evaluate the effect of particle size on the final mechanical and physical properties of the composites. The resin, hardener, and lunar regolith simulant were weighed into a beaker according to the desired formulations and mixed for approximately 10 min, taking care to minimize air entrapment. Once a homogeneous mixture was obtained, it was cast into silicone molds of different geometries: a plank-shaped mold for flexural tests (1 × 1 × 10 cm^3^), a disk-shaped mold for tribological tests (5 cm diameter × 0.5 cm height), and a cylindrical mold for tomography tests (1 cm diameter × 1 cm height) (Figure 1). The samples were left in the molds under ambient conditions for 24 h, then demolded and post-cured for 3 h at 100 °C. Finally, the sample surfaces were leveled by lapping with sandpaper (600 grit).

### 2.3. Characterizations

#### 2.3.1. Regolith Characterization

Powder particle size distributions were evaluated using a Mastersizer 3000+ Lab (Malvern Panalytical Ltd., Malvern, UK). Analyses were performed without any pretreatment, both before and after the sieving process.

X-ray diffraction (XRD) patterns of the lunar regolith simulant, before and after sieving, were acquired with an Anton Paar diffractometer (model XRDynamic 500, Anton Paar, Graz, Austria). The powders were placed in sample holders forming a uniform layer, and the holders were kept in rotation during measurement. Diffraction patterns were collected in the 20–60° 2θ range, using Bragg–Brentano geometry, with a step size of 0.03° and an acquisition time of 10 s per step.

#### 2.3.2. Composites Characterization

Mechanical characterization of the composites was carried out with a Universal Testing Machine (Instron, Norwood, MA, USA, Model 68TM-50), equipped with static load cells of 10 kN and a three-point flexure fixture with a span of 64.0 mm. Tests were conducted according to ASTM D790 (2017): Standard Test Method for Flexural Properties of Unreinforced and Reinforced Plastics and Electrical Insulating Materials [18].

Surface hardness of the composites was determined using a portable double-needle Shore D hardness tester (Borletti, Antegnate (BG), Italy). Tests were performed by applying a 44.5 N force with a 30° conical indenter. Hardness values were recorded immediately after force application and after 5 s. Each measurement was repeated five times, and average values were reported.

X-ray microtomography (µCT) analyses were performed using an EasyTom L system (RX Solutions, Chavanod, France). The source was a Hamamatsu microfocus X-ray tube operated at 150 kV with a tungsten target and a power of 17 W (111 µA). Projections were acquired with a Vieworks flat panel detector (1268 × 1012 pixels, 0.119 µm pixel size). Data acquisition was carried out in stack mode with continuous rotation and reference image correction enabled. The dataset consisted of six runs, each comprising 704 images, with an average of nine frames per projection. The final reconstructed voxel size was 17.25 µm.

Particle speciation analyses were performed using a Hitachi SU8700-VP Field-Emission Scanning Electron Microscope (FE-SEM) equipped with an Oxford Instruments UltimMax 40 Silicon Drift Detector (SDD) Energy Dispersive Spectrometer (EDS) and the feature analysis software package. The system operated at an acceleration voltage of 20 kV and a nominal beam current of 1.2 nA. SEM-BSE (back-scattered electron) images were acquired on finely ground samples to obtain micrographs suitable for automated particle analysis. The feature software identified particles based on grayscale contrast, excluding the polymer matrix and particles smaller than a specific threshold (5 µm for all samples, except for the finest fraction, where the threshold was reduced to 2 µm). For each identified particle, size and shape parameters were retrieved, followed by chemical characterization via EDS. Spectra were acquired with a live time of 5 s per particle; compositions were converted into oxide weight percentages (wt.%) and particles were classified according to their chemistry. Results are reported as volume percentages, calculated based on the cumulative areas of the basalt and anorthosite particles.

Sample images were acquired with a digital magnifier (OCULUX Macro Zoom, Microconsult, Sesto Fiorentino (FI), Italy). This system provides images with diffuse and uniform illumination over the entire sample surface, regardless of whether the geometry is concave, convex, flat, spherical, angular, or irregular.

Scanning Electron Microscopy (SEM) images were obtained with a Hitachi S-2300 instrument (Hitachi, Tokyo, Japan), operating at an accelerating voltage of 15 keV and a magnification factor of 100×.

Wear resistance was evaluated by ball-on-disk tribometry (Ducom, model POD.4, Ducom Instruments Inc., Bohemia, NY, USA). A 6 mm diameter alumina ball was used as the stationary counterpart, under a load of 20 N and a sliding speed of 0.16 m/s. The wear track diameter was set to 30 mm. Tests were performed until a total sliding distance of 70 m was reached.

The morphology of the wear tracks was characterized using a Keyence VK-3000 profilometer (Keyence, Osaka, Japan), based on confocal laser scanning microscopy, with a 50× zoom (5× optical lens combined with an internal 10× zoom). Images were analyzed with the dedicated VK-X3000 MultiFileAnalyzer software (version 3.3.1.85).

## 3. Results and Discussions

### 3.1. Composite Preparation

**Figure 2:**
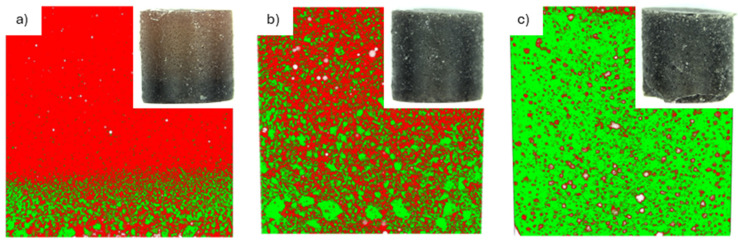
X-ray microtomography cross-sections of the composite samples containing different loads of lunar regolith simulant: (**a**) 30 wt.%, (**b**) 60 wt.%, and (**c**) 70 wt.%. The different phases are color-coded: red represents the resin, green the regolith, and white the voids. The images display the full cross-section of the samples along their diameter (approx. 1 cm), providing a dimensional reference. Insets show macroscopic photographs of the specimens.

To determine the optimal loading of lunar regolith simulant within the epoxy resin, three formulations (30, 60, and 70 wt.%) were initially compared. This selection was based on a preliminary optimization phase where alumina powder was used as a surrogate filler (to preserve the regolith simulant for the final characterization). In these preliminary tests, systematic screening with smaller concentration increments was performed, identifying 60 wt.% as the maximum filler content that ensures an optimal balance between high loading and processability. The suitability of the regolith/resin ratio was assessed by considering both rheological and mechanical properties. In particular, the castability of the uncured mixtures and the resulting internal microstructure were evaluated through microtomography cross-sectional analyses (Figure 2). The microtomography analysis allowed for the quantification of morphology, distribution, volume fraction, and the connectivity of the segmented phases. As reported in the literature, by quantifying manufacturing defects (e.g., void volume fraction), quantitative X-ray CT analysis can be applied to composites to identify optimal processing conditions as well as to assess the extent of damage (e.g., the number of matrix cracks) [19].

From the microtomography analysis (Figure 2), it is evident that the regolith distribution within the matrix is non-uniform at low filler contents (30 wt%). Specifically, regolith segregation by size can be observed, suggesting that sedimentation or phase separation occurred during the preparation or compaction process. It is important to note that these tests do not account for the reduced gravity on the Moon, which would naturally limit the visible sedimentation effect observed under terrestrial gravity. Nevertheless, the formation of such a heterogeneous distribution of solid reinforcement is unacceptable for practical applications. However, this sedimentation effect is effectively overcome when the dispersed material content reaches 60 wt.% or higher. At these higher loadings, the physical crowding and steric hindrance of the particles themselves prevent them from settling.

Conversely, a loading of 70 wt.% resulted in a more uniform particle distribution but also in a high void fraction and a marked reduction in castability, as the mixture became excessively thick and viscous. This effect is illustrated in Figure 2, where the 70 wt.% specimen fails to retain its expected cylindrical geometry, exhibiting imperfections at the bottom. In comparison, the composite with 60 wt.% displayed both good particle distribution (free from sedimentation) and satisfactory castability. Therefore, this formulation was identified as the best compromise to maximize the amount of usable regolith and the overall material performance.

The influence of particle size distribution on the mechanical properties of the composite systems was subsequently investigated. Four fractions of the lunar regolith simulant were obtained by sieving, as follows:

- Particles larger than 200 µm;
- Particles between 200 µm and 100 µm;
- Particles between 100 µm and 50 µm;
- Particles smaller than 50 µm.

The resulting fractions were collected in relative proportions of 1:2:2:1, indicating a symmetric distribution centered on the intermediate size ranges.

**Figure 3:**
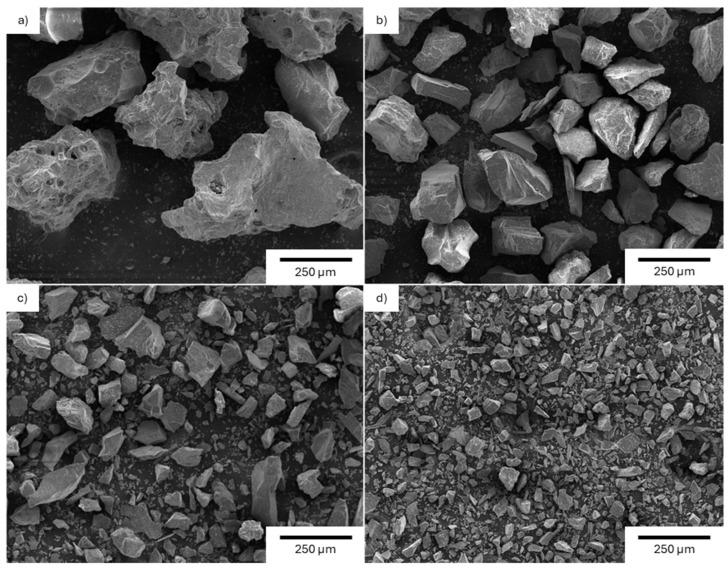
SEM images of sieved lunar regolith simulant: (**a**) particles with dimensions higher than 200 µm; (**b**) particles with dimensions between 200 µm and 100 µm; (**c**) particles with dimensions between 100 µm and 50 µm, and (**d**) particles with dimensions lower than 50 µm.

**Figure 4:**
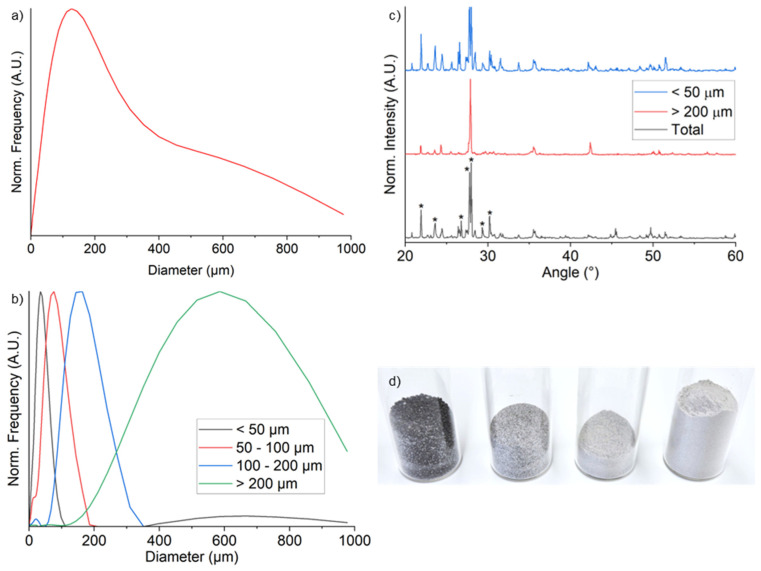
(**a**,**b**) show the particle size distribution of the lunar regolith simulant powder respectively before and after the sieving process; (**c**) XRD analysis of the lunar regolith simulant before and after the sieving process (* refers to triclinic anorthite diffraction peaks, ICDD-ICDD PDF-2 41-1486); (**d**) image of the sieved fraction of lunar regolith simulant (from left to right: particles with dimensions higher than 200 µm, particles with dimensions between 200 µm and 100 µm, particles with dimensions between 100 µm and 50 µm, and particles with dimensions lower than 50 µm).

Besides the obvious decrease in particle size due to the sieving process, the SEM images in Figure 3 confirm that there are no significant differences in particle morphology among the fractions while the particle size distribution analysis (Figure 4a,b) confirms the narrow bell shape of the four fractions with a slight overlap between adjacent fractions.

Figure 4c shows the XRD spectra of the unsieved sample and of the <50 and >200 fractions. As stated in the product technical specification sheet, the unsieved sample mainly exhibits diffraction peaks related to anorthosite (≈75 wt.%, marked with stars in the diffraction pattern), while the remaining ≈25 wt.% consists of glass-rich basalt. After sieving, the largest fraction (>200 µm) appeared darker in color compared to both the unsieved sample and the other fractions (Figure 4d) suggesting the latter contains a larger fraction of basalt. This interpretation is supported by the XRD patterns in Figure 4c, where the typical anorthosite doublet at 28° is barely detectable in the >200 µm fraction.

**Figure 5:**
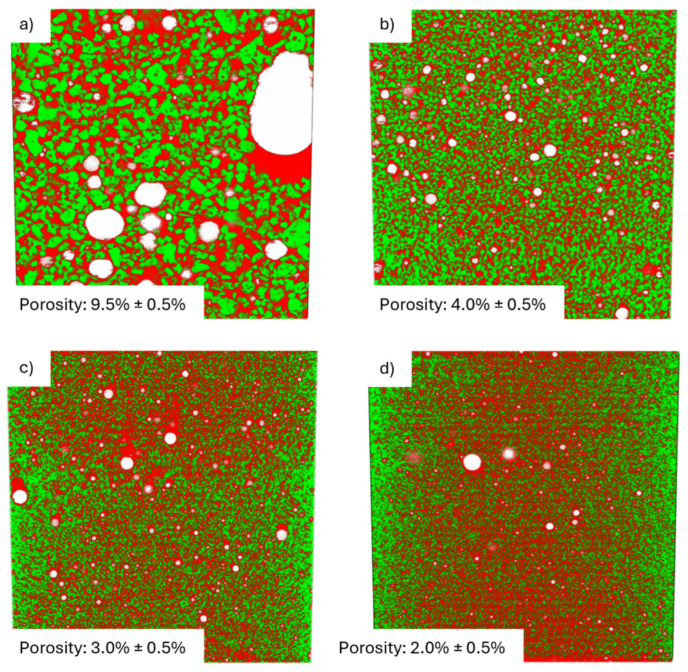
Microtomography cross-sections of the composites obtained starting from different size distribution fractions of lunar regolith simulant: (**a**) Particles with dimensions higher than 200 µm; (**b**) particles with dimensions between 200 µm and 100 µm; (**c**) particles with dimensions between 100 µm and 50 µm; and (**d**) particles with dimensions lower than 50 µm. Red indicates the epoxy resin, green the regolith, and white the empty spaces. Inset: porosity percentage of the samples.

The effect of particle size on the composite structure was evaluated through the acquisition and analysis of microtomography cross-sections (Figure 5).

The images show the uniform distribution of particles throughout the bulk of all samples. It is crucial to note that the apparent increase in green signals observed near the sample edges, particularly in the smaller-particle fractions, is purely an instrumental artifact [20,21]. This phenomenon, known as the beam hardening effect, should not be misinterpreted as a real enrichment or segregation of particles at the periphery, as confirmed by complementary cross-sectional observations.

Porosity was quantified using ImageJ software (version 1.54g) by applying a global threshold to the reconstructed microtomography stacks. To ensure statistical significance, the total volume of each sample was divided into five sub-volumes; the mean porosity and its corresponding standard deviation were then calculated based on these measurements.

**Figure 6:**
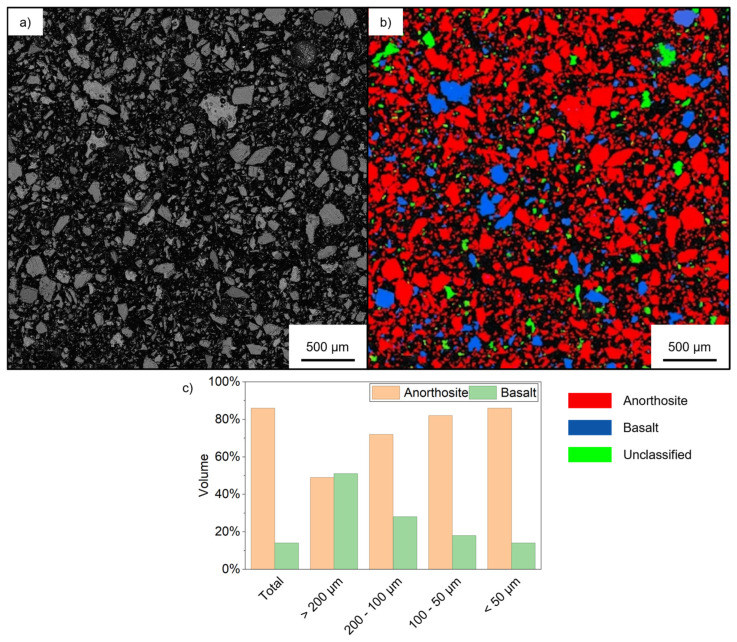
(**a**) SEM-BSE image of the composite obtained with unsieved regolith simulant powder, (**b**) the speciated particles obtained after SEM-EDS analysis, and (**c**) the volume percentage of anorthosite and basalt for the composite samples obtained with sieved lunar regolith simulant compared to those obtained with unsieved material.

To verify the basalt enrichment in the particle fraction coarser than 200 µm, SEM-EDS measurements were performed on sample sections. With explicative purpose, Figure 6 depicts the SEM-BSE image of the composite obtained with the unsieved regolith simulant powder (Figure 6a), together with speciated particle image (Figure 6b). Similar images were obtained for all the other samples. The results of the speciation, depicted in Figure 6c, confirm that basalt particles are larger than anorthosite ones. This size disparity leads to the basalt enrichment observed in the composite produced with sieved particles >200 µm. This enrichment decreases as particle size diminishes, with the finest fraction reaching values nearly identical to those of the unsieved sample. The quantitative EDS data are in good agreement with the XRD peak intensity variations, confirming that the lighter color and reduced mechanical performance of the finest fraction are indeed linked to a lower basalt concentration.

### 3.2. Mechanical Characterization

**Figure 7:**
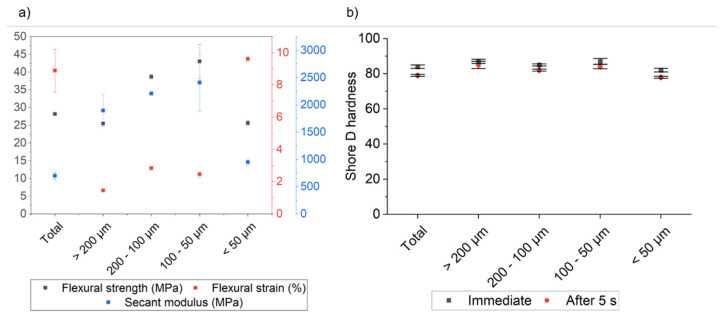
(**a**) Flexural strength, flexural strain, and secant modulus value, and (**b**) Shore D hardness values in function of the distribution size of the lunar regolith simulant particles.

Figure 7a shows the flexural strength, flexural strain and secant modulus values of the composites as a function of the lunar regolith simulant particle size distribution. The first values (indicated as “Total”) correspond to the composite prepared with unsieved filler material.

A marked decrease in flexural strain was observed for the sample containing larger particles, suggesting increased material rigidity. Nevertheless, the flexural strength remained comparable, likely because of the weakening effect induced by the higher porosity revealed by microtomography.

Both samples containing particles in the ranges of 200–100 µm and 100–50 µm exhibit flexural strain values like the sample with larger particles, but with higher flexural strength. This behavior confirms that the presence of particles in the composites increases not only rigidity but also resistance to flexural stress [22,23,24].

By contrast, the sample obtained with the smallest particles exhibits a reduction in flexural strength and an increase in flexural strain, thereby losing the improvements observed in the other samples. This behavior can be attributed to the lower content of glass-rich basalt in this fraction—as confirmed by XRD analysis and by the lighter color of the sieved powder (basalt, compared with anorthosite, is the harder component of lunar regolith)—together with the small particle size, which may limit the reinforcing effect within the composite material.

Flexural modulus presents a different behavior: the composite material reinforced with the unsieved particles shows a value of about 700 MPa. After sieving the composite material reinforced with the biggest particles, the modulus increases to about 1900 MPa and remains high, with sieved particles with dimensions between 200 µm and 100 µm and between 100 µm and 50 µm. The composite material reinforced with the smallest particles again presents a decrease in the observed value, confirming the trends observed for the other parameters, and is probably explainable by the same considerations.

Considering both the basalt enrichment evaluated via SEM-EDS and the mechanical properties of the composites, it is noteworthy that the unsieved sample exhibits nearly the same properties and basalt/anorthosite ratios as the fraction with the smallest particles. Consequently, the observed variations in mechanical properties can be primarily attributed to changes in the basalt/anorthosite fractions; however, a minor influence from particle size cannot be entirely ruled out.

These results are consistent with the measured Shore D hardness values reported in Figure 7b, which show a slight decrease in hardness for the sample prepared with the smallest particles, returning to a level comparable to that of the composite produced using unsieved powder.

**Figure 8:**
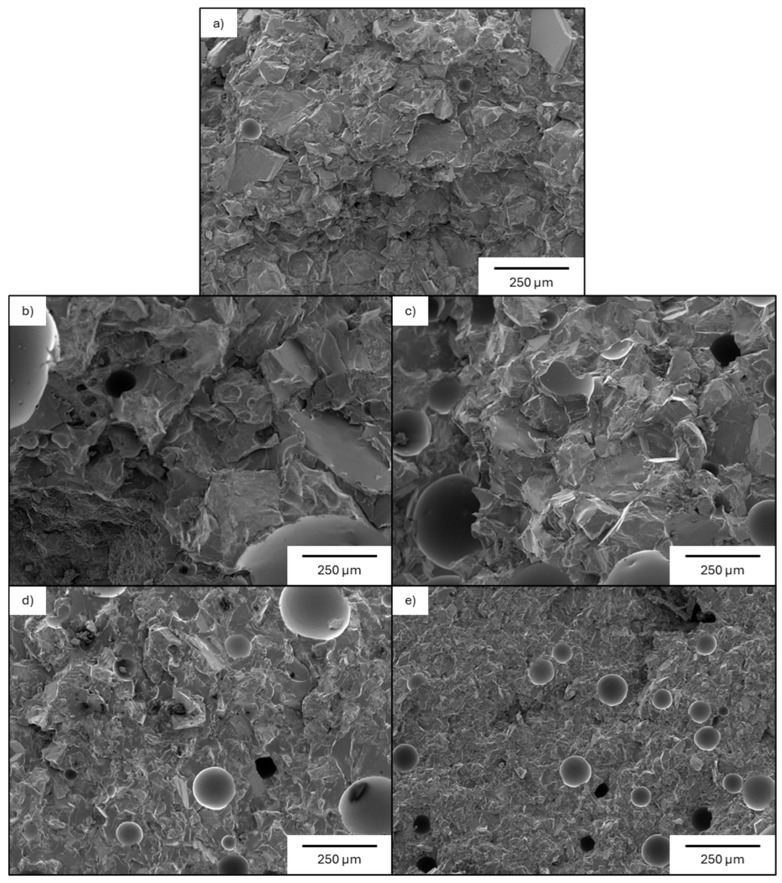
SEM images of the fracture of the composites obtained starting from different size distribution fractions of lunar regolith simulant: (**a**) Unsieved particles, (**b**) particles with dimensions higher than 200 µm; (**c**) particles with dimensions between 200 µm and 100 µm; (**d**) particles with dimensions between 100 µm and 50 µm; and (**e**) particles with dimensions lower than 50 µm. Red indicates the epoxy resin, green the regolith and white the empty spaces.

The fracture cross-section SEM images in Figure 8 describe the regolith-based epoxy composite characterized by a relatively compact microstructure with few sharp edges and a smooth surface almost free of prominent cracks and gaps between particles and resin. The fracture morphology is consistent with high strength material exhibiting brittle fracture behavior.

**Figure 9:**
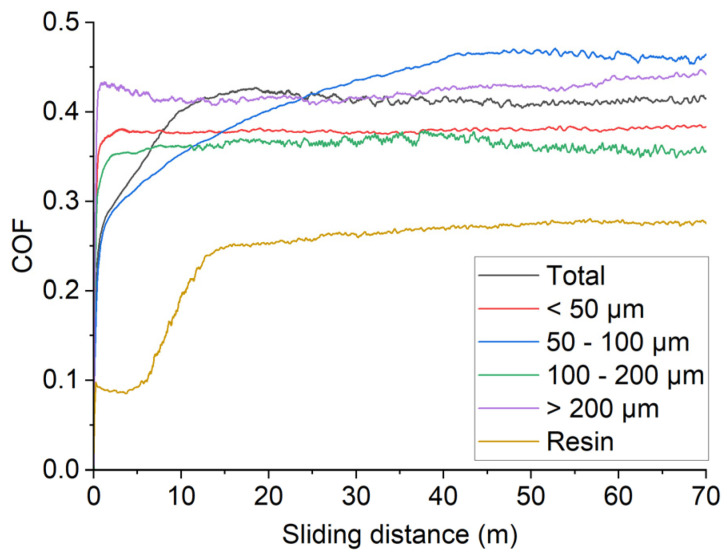
COF values in function of sliding distance for composite samples obtained with sieved lunar regolith simulant compared to that obtained with unsieved material.

Figure 9 reports the COF values as a function of the sliding distance for each sample compared to the resin one. The sample set comprised four specimens prepared with sieved lunar regolith simulant and one prepared using unsieved powder. The coefficients of friction (COF) for all samples were in the range of approximately 0.35–0.45, with no significant differences observed among them. This behavior can be attributed to the composition of the composites: all were prepared with the same resin-to-particle ratio, resulting in the same amount of resin across the samples. During sliding, the resin may act as a lubricant—as suggested by the lower COF measured for the resin-only sample—resulting in comparable COF values [25]. Noticeable differences are limited to the first 10 m of sliding, likely due to slight differences in surface preparation.

**Figure 10:**
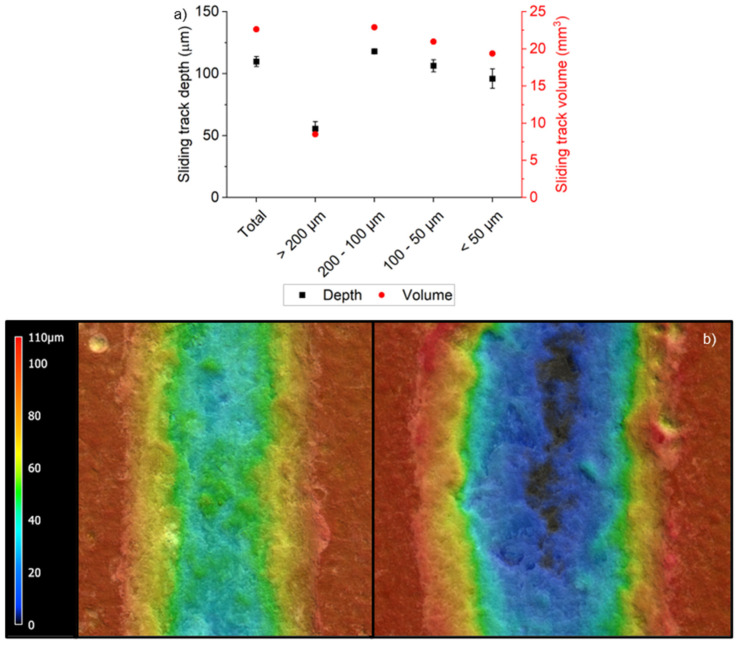
(**a**) Sliding track depth and volume as function of the distribution size of particles and (**b**) optical profilometry images of the sliding track on the sample obtained with the sieved particles with dimensions higher than 200 µm (**left**) compared to that of the sample obtained with unsieved particles.

Figure 10 presents both the wear tracks depth and their volumes as a function of the particle size distribution of the regolith simulant, compared with the sample produced from unsieved powder. Both parameters display a similar overall trend across the samples. No significant differences were observed between the sample obtained with unsieved particles and those obtained with fine particles (<200 µm). In contrast, the sample prepared with larger particles (>200 µm) exhibited markedly lower wear track depth and volume, indicating a beneficial reinforcement effect of coarse particles on wear resistance. Both particle size and composition are likely to contribute to this effect: larger particles provide more effective reinforcement against wear than smaller ones, and the higher content of glass-rich basalt in this powder fraction further enhances the composite performance. These results are consistent with the optical profilometry images shown in Figure 10, which illustrate, as examples, the wear tracks of the sample prepared with particles larger than 200 µm, and the sample obtained with unsieved powder.

To distinguish between the influence of particle size and mineralogical composition, the performance of the unsieved composite was compared to that of the finest fraction (<50 µm). Despite the significant difference in their particle size distributions, SEM-EDS speciation showed that both samples possess a similar basalt/anorthosite ratio. The fact that they exhibit nearly equivalent flexural strength and wear resistance suggests that basalt enrichment is the dominant factor governing the mechanical enhancement. If particle size were the primary driver, a significant discrepancy between these two formulations would have been expected. Therefore, the superior performance of the coarsest fraction (>200 µm) can be primarily attributed to its higher basalt content, with particle size providing only a minor synergistic contribution.

## 4. Conclusions

This study highlighted the critical role of particle size distribution in determining the mechanical and functional performance of epoxy–regolith composites for mold-casting applications. A filler loading of 60 wt.% was identified as the optimal formulation, balancing castability and regolith content. Sieving the regolith simulant revealed that:

- large particles (>200 µm) promote high porosity but enhance wear resistance.
- intermediate fractions (200–100 µm and 100–50 µm) improve flexural strength while maintaining good material integrity.
- fine particles (<50 µm) reduce reinforcement efficiency due to both their size and lower basalt content.

Tribological results indicate that the coefficient of friction is dominated by the resin matrix, whereas particle size governs wear depth and volume.

These findings underline the importance of tailoring particle size and composition in regolith-based composites for In Situ Resource Utilization (ISRU) applications. Optimizing granulometry can significantly enhance mechanical performance and durability, thus contributing to the development of reliable and locally sourced composites for the on-site manufacturing of non-structural, everyday items and functional tools within future lunar outposts, ultimately reducing the payload reliance on Earth.

## Figures and Tables

**Table 1:** Samples Composition for the Preliminary Phase.

Sample ID	Total Resin Weight (g)	Resin Weight (g)	Hardner Weight (g)	Regolith Weight (g)	Regolith wt.%
R/r 70/30	35.0	25.0	10.0	15.0	30 wt.%
R/r 40/60	26.6	19.0	7.6	40.0	60 wt.%
R/r 30/70	19.3	13.8	5.5	45.0	70 wt.%

## Data Availability

The original contributions presented in this study are included in the article. Further inquiries can be directed to the corresponding authors.
